# Assessing the Impacts of Integrated Decision Support Software on Sexual Orientation Recording, Comprehensive Sexual Health Testing, and Detection of Infections Among Gay and Bisexual Men Attending General Practice: Observational Study

**DOI:** 10.2196/10808

**Published:** 2018-11-06

**Authors:** Denton Callander, Christopher Bourne, Handan Wand, Mark Stoové, Jane S Hocking, John de Wit, John M Kaldor, Basil Donovan, Catherine Pell, Robert Finlayson, David Baker, Bradley Forssman, BK Tee, Bill Kefalas, Tim Duck, Rebecca Guy

**Affiliations:** 1 The Kirby Institute University of New South Wales Sydney Sydney, NSW Australia; 2 Centre for Social Research in Health University of New South Wales Sydney Sydney, NSW Australia; 3 Sydney Sexual Health Centre Sydney Hospital Sydney, NSW Australia; 4 Sexually Tranmissible Infection Programs Unit New South Wales Health Sydney, NSW Australia; 5 Burnet Institute Melbourne, VIC Australia; 6 School of Public Health and Preventative Medicine Monash University Melbourne, VIC Australia; 7 Melbourne School of Population and Global Health Melbourne University Melbourne, VIC Australia; 8 Taylor Square Private Clinic Sydney, NSW Australia; 9 East Sydney Doctors Sydney, NSW Australia; 10 Fountain Street General Practice Sydney, NSW Australia; 11 The Centre Clinic Victorian AIDS Council Melbourne, VIC Australia; 12 University of New South Wales Health Service University of New South Wales Sydney Sydney, NSW Australia; 13 New South Wales Ministry of Health Sydney, NSW Australia

**Keywords:** men who have sex with men, general practice, sexual health, software, STI

## Abstract

**Background:**

Gay and bisexual men are disproportionately affected by HIV and other sexually transmissible infections (STIs), yet opportunities for sexual health testing of this population are often missed or incomplete in general practice settings. Strategies are needed for improving the uptake and completeness of sexual health testing in this setting.

**Objectives:**

The goal of the research was to evaluate the impact of an intervention centered around integrated decision support software and routine data feedback on the collection of sexual orientation data and sexual health testing among gay and bisexual men attending general practice.

**Methods:**

A study using before/after and intervention/comparison methods was undertaken to assess the intervention’s impact in 7 purposively sampled Australian general practice clinics located near the urban centers of Sydney and Melbourne. The software was introduced at staggered points between April and August 2012; it used patient records to prompt clinicians to record sexual orientation and accessed pathology testing history to generate prompts when sexual health testing was overdue or incomplete. The software also had a function for querying patient management system databases in order to generate de-identified data extracts, which were used to report regularly to participating clinicians. We calculated summary rate ratios (SRRs) based on quarterly trends and used Poisson regression analyses to assess differences between the 12-month preintervention and 24-month intervention periods as well as between the intervention sites and 4 similar comparison sites that did not receive the intervention.

**Results:**

Among 32,276 male patients attending intervention clinics, sexual orientation recording increased 19% (from 3213/6909 [46.50%] to 5136/9110 [56.38%]) during the intervention period (SRR 1.10, 95% CI 1.04-1.11, *P*<.001) while comprehensive sexual health testing increased by 89% (305/1159 [26.32%] to 690/1413 [48.83%]; SRR 1.38, 95% CI 1.28-1.46, *P*<.001). Comprehensive testing increased slightly among the 7290 gay and bisexual men attending comparison sites, but the increase was comparatively greater in clinics that received the intervention (SRR 1.12, 95% CI 1.10-1.14, *P*<.001). In clinics that received the intervention, there was also an increase in detection of chlamydia and gonorrhea that was not observed in the comparison sites.

**Conclusions:**

Integrated decision support software and data feedback were associated with modest increases in sexual orientation recording, comprehensive testing among gay and bisexual men, and the detection of STIs. Tests for and detection of chlamydia and gonorrhea were the most dramatically impacted. Decision support software can be used to enhance the delivery of sexual health care in general practice.

## Introduction

In most high-income settings, the prevalence of HIV and other sexually transmissible infections (STIs) is high among gay, bisexual, and other men who have sex with men [1-3]. Combatting this disproportionate burden requires, among other strategies, routine and frequent sexual health testing, particularly among men whose sexual practices place them at risk of infection [4]. For this reason, clinical guidelines in countries like Australia, Canada, and the United States recommend that sexually active gay and bisexual men should receive a comprehensive sexual health screen at least once per year and more frequently as dictated by sexual risk [5-7].

Australian guidelines during this study defined a comprehensive screen for gay and bisexual men as one that involved tests for rectal and urogenital chlamydia, rectal and pharyngeal gonorrhea, infectious syphilis, and (among men not known to be infected), HIV [8]. The importance of comprehensive testing has been underscored by previous research, with one study finding that 60% of gonorrhea infections and 80% of chlamydia in gay and bisexual men would be missed if rectal swabs were not collected [9]. And although approximately three-quarters of gay men in Australia receive some form of sexual health testing annually, far fewer (37%) report receiving a comprehensive screen [10].

In many countries, general practice is responsible for a large amount of sexual health testing. In Australia, just over half of gay and bisexual men report receiving sexual health testing from general practice clinics [11,12] and the regularity with which people attend general practices makes them ideal for routine testing. Some general practitioners, however, are uncomfortable discussing issues of sexuality with patients or simply forget to raise sexual health due to a focus on the primary reason for presentation and other competing demands [13,14]. Further, studies have found that general practitioners rarely take patients’ sexual histories or record their sexual orientation [15-17], which is vital information for guiding any approach to sexual health care. Collectively, these factors may challenge the quality and completeness of sexual health care to gay and bisexual men attending general practice clinics. This contention is supported by research that found gaps in the uptake of sexual health testing among gay and bisexual men attending Australian general practice clinics [18].

One way to enhance health care provision is through computerized clinical decision support systems. Systems that prompt clinicians or provide them with tools to make clinical decisions have been shown in diverse fields of health to improve patient outcomes [19]. While a few studies have shown that clinician prompts can improve rates of testing for HIV and other STIs [20-22], nearly all have been based in sexual health clinics and focused on offering sexual health testing to all patients or all members of a particular population. In general practice, patients tend to be seen frequently and for diverse reasons [23], so any decision support system must consider an individual’s sexual risk and testing history.

The aim of this paper was to assess if decision support software can improve the delivery of sexual health care in general practice. To that end, we designed and implemented a computerized clinical decision support system that aimed to improve the recording of patient sexual orientation and promote comprehensive sexual health testing among gay and bisexual men. This paper evaluates the clinical impacts of this intervention, known as The eTEST Project.

## Methods

### Study Design

To assess the intervention’s impact, we undertook a quantitative observational study design involving before-after time series analyses at sites that received the intervention and assessment of concurrent trends between intervention and comparison clinics.

### Study Sites and Their Patients

#### Intervention Sites

Purposive sampling was undertaken to recruit 7 general practice clinics with minimum annual caseloads of 50 individual gay and bisexual male patients. All practices were located in Sydney and Melbourne, urban centers with the largest populations of gay and bisexual men in Australia and where approximately half of men report receiving sexual health care in general practice [11,12]. This study was limited to urban centers for practical reasons, as recruiting and supporting clinics in regional and remote areas would have exceeded available funding. Clinics were identified for recruitment through consultation with organizations representing general practice, sexual health, HIV medicine, and the health of gay and bisexual men. We also located clinics in or around neighborhoods with high concentrations of same-sex partnered households using Australian census data [24] and by posting study advertisements in medical newsletters. Of note, no sites contacted us to participate, suggesting that advertisements were ineffective for recruiting clinics to this kind of intervention research.

Our scoping exercise identified 28 potential sites, which through consultation with research partners in sexual health and general practice was reduced to the 19 most likely to see reasonably sized caseloads of gay and bisexual men. Potential sites were sent an introductory letter or email that outlined the study and proposed an in-person meeting. After introductory information was sent, 12 in-person meetings were undertaken. From those meetings, 3 clinics were found to not have sufficient numbers of gay and bisexual male patients, resulting in 9 sites recruited to participate. Two sites withdrew participation because their practice computers did not meet the minimum requirements for installing and operating the software.

#### Comparison Sites

In addition to the intervention sites, a convenience sample of comparison sites was created by extracting data from the patient management systems of clinics based in urban areas of Sydney and Melbourne. We selected comparison sites because it was possible to extract data from their patient management systems and they bore similarities to the intervention clinics, each with a minimum of 50 individual gay and bisexual men seen annually and located within 10 kilometers of the intervention sites. Comparison sites were identified as potential intervention clinics but ultimately did not receive the intervention either because they were not interested or their patient management system was incompatible with the intervention software. The number of intervention sites was limited given the general rarity of “gay” general practice clinics of this kind in Sydney and Melbourne.

### Study Intervention

We designed a computerized clinical decision support system for sexual health in general practice. The software built upon an existing piece of technology used in general practice known as the PrimaryCare Sidebar, which worked by querying patient databases and using those queries to generate prompts, produce assessments, or trigger patient recalls. One of the 7 study sites used a patient management system incompatible with the study software and, therefore, participated using a modified version of the intervention that involved establishing prompts using existing built-in assessment tools.

A sexual health-specific module was added to the existing Sidebar software. The module included a custom-built sexual history tool that facilitated the assessment and recording of sexual risk practices for gay and bisexual male patients. The tool routed patients into 3 simple categories linked to testing frequency recommendations: high risk (testing every 3 to 6 months: ≥10 sexual partners in the past 6 months or condomless anal sex with a casual partner), medium risk (annual testing: any anal sex in past 6 months), and low risk (test as needed: no sex in past year or in a monogamous relationship). The assessment dialogue also included links to online sexual health testing guidelines [25] and a partner notification resource to which patients could be referred [26].

**Figure 1:**
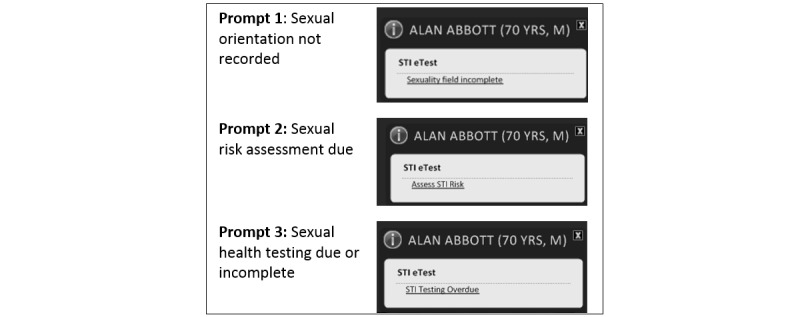
Record-generated, passive prompt dialogues in a sexual health–specific module for decision support software in general practice that encourage clinicians to record patient sexual orientation, collect a sexual risk assessment, and conduct sexual health testing.

Additionally, the sexual health module of Sidebar included a system of electronic prompts (Figure 1). These prompts were triggered by opening of the patient records and were dynamic but passive in that they appeared after a patient record was opened and faded shortly thereafter. The fading function was specifically requested by participating doctors in order to reduce unnecessary interference with consultations. Sexual health prompts were activated by the following events:

- Sexual orientation details were not included in a patient’s record.
- A risk level assessment had not been recorded for a gay or bisexual patient.
- Sexual health testing for a gay or bisexual patient was due or incomplete.

Prompts for HIV and STI testing were triggered on the basis of pathology records in the patient management system, which were automatically downloaded from servicing laboratories. Prompts for testing were also generated depending on assessed risk, with clinicians prompted 6 months after previous testing for patients assessed as high risk and after 12 months for medium-risk patients. Of note, prompts were only generated if a patient’s electronic record were opened. Following a 6-month pilot at one clinic, staggered implementation of the software occurred between April and August 2012 and operated in each clinic for a minimum of 24 months with clinics finishing between April 2014 and August 2014. An overview of the prompt system and risk assessment dialogue is provided as a video in Multimedia Appendix 1.

Beyond the user-facing component, the intervention software also facilitated routine extraction of de-identified data from male patients attending each service. To do so, the software would query the patient database to generate a comma-separated values table based on a customized schema for data extraction. The table included line-listed patient data but without any identifiers, such as address or name. These data were used to evaluate the software but also included as part of the intervention itself. Specifically, clinics were provided biannual reports on testing trends, test positivity, and sexual orientation recording at their service. These reports were routinely presented at clinic meetings to allow doctors a chance to discuss the data, ask questions, and share feedback on the software itself. Individually, clinicians also received tailored emails that focused on different indicators specific to their patients and with comparisons within and between clinics. In these ways, clinical data were used like a model of quality improvement.

### Intervention Impact

#### Evaluation Pathway

**Figure 2:**
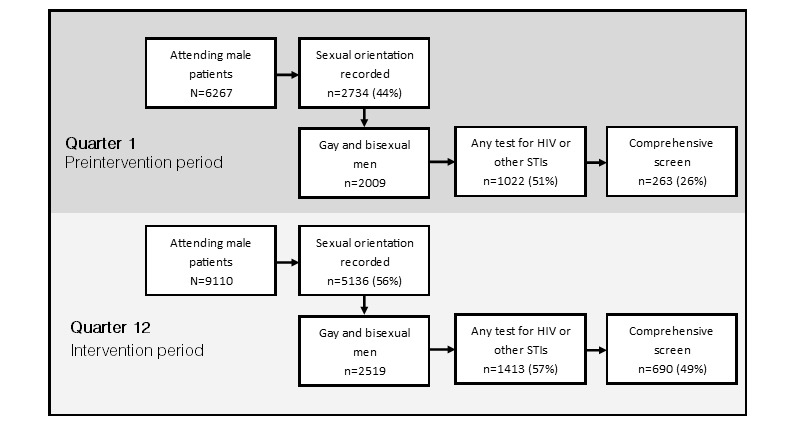
Clinical pathway for assessing intervention impact between the pre- and intervention periods among sites that received the intervention. STI: sexually transmissible infection.

The intervention’s impact was assessed along a clinical pathway of 3 outcome variables: sexual orientation recording, HIV and/or STI testing uptake, and comprehensive sexual health testing. Figure 2 provides an overview of this pathway. As noted, the intervention was introduced at staggered points throughout 2012, which required us to organize the study period into 12 quarters: preintervention period (quarters 1 through 4) and intervention period (quarters 5 through 12). Comparison sites were also organized into quarters, which were established at the mean entry point for the intervention sites, meaning that quarter 1 for comparison sites began in June 2012 and quarter 12 ended in May 2014. Stata version 14 (StataCorp LLC) was used for all analyses.

#### Sexual Orientation

Sexual orientation recording was calculated as the proportion of attending male patients for whom sexual orientation details were collected. To assess changes over time, we calculated quarterly trends in the proportion of male patients with recorded sexual orientation in the pre- and intervention periods via summary rate ratios (SRRs). SRRs are useful for assessing relative differences in an event occurring over a fixed time frame, which we compared between the pre- and intervention periods using Poisson regression analysis. Unfortunately, differences in how data were extracted between intervention and comparison sites meant that it was not possible to compare changes in sexual orientation between these two clinical groups.

#### Uptake of Testing for HIV and Sexually Transmissible Infections

The second indicator of the intervention’s impact focused on the proportion of men recorded as gay and bisexual men who had any test for HIV or other STI. As with sexual orientation, we calculated this on a quarterly basis and calculated the SRR for the pre- and intervention periods, which were compared using a Poisson regression analysis. Changes over time were also assessed between intervention and comparison sites using a Poisson regression analysis.

In order to facilitate comparisons over time, it was necessary to apply patient sexual orientation across the entire study period. This means that if a patient was later recorded as gay or bisexual we categorized him as such for the purposes of calculating these indicators. It is also important to note that because sexual orientation recording was very low among men attending clinics in the comparison group, to facilitate comparison we used a history of rectal swabs for STI testing to identify patients likely to be gay or bisexual. This has been shown previously to be an effective proximal marker for this population [27], which while not ideal was a necessity for comparative purposes.

#### Comprehensive Sexual Health Testing

Comprehensive sexual health testing was calculated as the proportion of gay and bisexual men who received any HIV or STI test—chlamydia (rectal or urogenital), gonorrhea (rectal or pharyngeal), syphilis and, among men not known to be infected, HIV. SRRs were calculated and differences over time and between intervention and comparison sites were assessed.

#### Detection of HIV and Other Sexually Transmissible Infections

Finally, we assessed changes to the detection of HIV and other STIs using positive pathology or, for infectious syphilis, by reviewing historical test results and the interpretative comments provided by labs. In situations where infectious syphilis could not be determined (ie, insufficient information), the result was excluded. SRRs were calculated to assess the mean number of infections diagnosed quarterly during the pre- and intervention periods, which were compared using a Poisson regression analysis. A similar analysis was conducted to assess differences in detection of infections between the intervention and comparison sites.

### Data Sources

We collected patient data using a data extraction component of the intervention software. De-identified, line-listed patient data were extracted for the 12-month pre- and 24-month intervention periods. For male patients aged 14 years and older, we extracted the following details per patient visit: unique identifier, age, home postcode, Indigenous status, sexual orientation, HIV status, visit date, visit reason, provider, and HIV/STI testing conducted. Because the results of pathology were downloaded into patient management systems as free text, they could not be extracted directly from participating clinics. Thus, parallel HIV and STI pathology results for all male patients were extracted from the laboratories that serviced participating sites.

Data for comparison sites were extracted from an existing sentinel surveillance network for bloodborne viruses and STIs. The Australian Collaboration for Coordinated Enhanced Sentinel Surveillance (ACCESS) routinely extracts de-identified patient data from a range of clinical sites across Australia and provided the comparison data for sites not participating in the study intervention. Details on this project have been published previously [28].

### Ethical Review

Ethical review of this study was provided by the University of New South Wales Human Research Ethics Committee (HC10310). Informed consent was obtained from general practitioners based at clinics that received the intervention.

## Results

### Study Sites and Their Patients

#### Intervention Sites

In total, 7 general practices participated in the study intervention, all of which were located in inner urban areas. Participating clinics employed between 3 and 17 general practitioners in full or part-time service. In total, 66 general practitioners participated in the intervention, of which 28 (42%) were female.

During the 3-year observation period, 32,276 individual male patients aged 14 years and older attended intervention clinics with a range of 1905 to 8711 patients per clinic. The median age at baseline was 46 years (interquartile range: 36-56 years), the majority of patients (24,007/32,276, 74.38%) were HIV negative and, reflecting the exclusively urban nature of participating sites, most patients (31,375/32,276, 97.21%) lived in major cities. Only 53.45% (17,251/32,276) of patients had Indigenous status included in their record, with 0.61% (196/32,276) recorded as being of Aboriginal or Torres Strait Islander background.

#### Comparison Sites

Four general practice clinics were identified as comparison sites, 2 based in Sydney and 2 in Melbourne. In total, 23,712 individual male patients attended comparison sites during the 24-month study period with a range of 952 to 10,279 male patients per clinic. Demographically, the comparison patient group was similar to those attending intervention sites with a median baseline age of 46 years (interquartile range: 36-56 years). In total, 75.41% (17,881/23,712) of male patients attending these clinics were HIV negative, 94.67% (22,448/23,712) lived in an urban area, and 1.67% (396/23,712) were of Aboriginal or Torres Strait Islander background.

### Intervention Impact

#### Sexual Orientation Recording

Figure 2 outlines the clinical pathway we used to evaluate this intervention, comparing the first quarter of the prestudy period (Q1) with the last quarter of the intervention period (Q12). In the first quarter, 43.63% (2734/6267) of attending male patients had details about their sexual orientation recorded, which remained stable across the prestudy period (2961/6639 [44.60%] in Q4). During the intervention period, however, the proportion of male patients with sexual orientation details increased from 46.50% (3213/6909) in Q5 to 56.38% (5136/9110) in Q12 (*P*<.001) with an SRR of the average trend between the before and intervention periods of 1.10 (95% CI 1.04-1.11, *P*<.001). In intervention sites, increases in recording of sexual orientation were observed across age groups, with the lowest baseline proportion but the greatest change in patients less than 30 years old, increasing by 71% during the intervention period from 17.06% (252/1477) recorded in Q5 to 29.45% (494/1680) in Q12 (*P*<.001).

Multimedia Appendix 2 provides an overview of the SRRs of sexual orientation recording between the before and intervention periods. As noted, it was not possible to calculate this variable among sites in the comparison group, noting that only 4.26% (1010/22,702) of men attending these clinics had sexual orientation included formally in their record. Using rectal swab details, however, it was possible to identify 30.74% of men attending these sites as either gay or bisexual (7290/23,712 of male patients).

#### Uptake of Testing for HIV or Sexually Transmissible Infections

As detailed in Figure 2, sexual orientation recording was only the first step in our clinical pathway. Among men recorded as gay or bisexual, we also assessed the proportion who in a quarter had any test for HIV or other STIs. In Q1, 50.87% (1022/2009) of attending gay and bisexual men received some form of sexual health testing, which was stable during the preintervention period (1115/2185 [51.03%] in Q4) and also during the intervention period (1159/2290 [50.61%] in Q5 to 1413/2519 [56.09%] in Q12, *P*=.9). There did not appear to be a difference in sexual health testing uptake between the pre- and intervention periods (SRR 0.97, 95% CI 0.94-1.00, *P*=.2) nor was a change observed in comparison sites (SRR 1.00, 95% CI 0.97-1.02, *P*=.9).

#### Comprehensive Sexual Health Testing

Among men who received a test for HIV or other STI, only 25.73% (263/1022) in Q1 went on to receive the full complement of tests recommended by guidelines. This proportion remained stable during the preintervention period (306/1115 [27.44%] in Q4, *P*=.5) but increased during the intervention period (305/1159 [26.32%] in Q5 to 690/1413 [48.83%] in Q12), representing an 88% relative increase in comprehensive testing (*P*<.001). The SRR comparing the quarterly before and intervention trends of comprehensive testing was 1.37 (95% CI 1.28-1.43, *P*<.001). Multimedia Appendix 2 provides an overview of the comparative increases in comprehensive sexual health testing among gay and bisexual men by HIV status and age. Increases in comprehensive testing were observed across age groups, including a relative increase of 84% increase among men 30 years and younger (17/46 [37%] to 93/137 [67.9%], *P*<.001), a 79% increase among men aged 30 to 49 years (174/596 [29.2%] to 417/796 [52.4%], *P*<.001), and a twofold increase among men aged 50 years and older (72/380 [19.0%] in Q5 to 180/480 [37.5%] in Q12, *P*<.001). Comprehensive testing doubled also among patients living with HIV (94/560 [16.8%] to 240/665 [36.1%], *P*<.001).

Tests for HIV and syphilis were, by far, the most common component of testing events among gay and bisexual patients. Prior to the intervention, 83.55% (12,016/14,282) of testing events included syphilis and HIV, which increased to 89.46% (29,934/33,461) for syphilis during the intervention period (*P*<.001) but remained stable for HIV (*P*=.1). The overall proportions of tests involving chlamydia and gonorrhea were much lower in the before period: 19.90% (2842/14,282) of testing events included rectal swabs for chlamydia and gonorrhea, which increased to 34.51% (11,547/33,461) during the intervention (*P*<.001), while urine testing for chlamydia increased from 22.00% (3142/14,282) to 37.67% (12,605/33,461) (*P*<.001). Pharyngeal swabs for gonorrhea also increased, from 17.13% (2447/14,282) before to 34.71% (11,614/33,461) during the intervention (*P*<.001).

**Figure 3:**
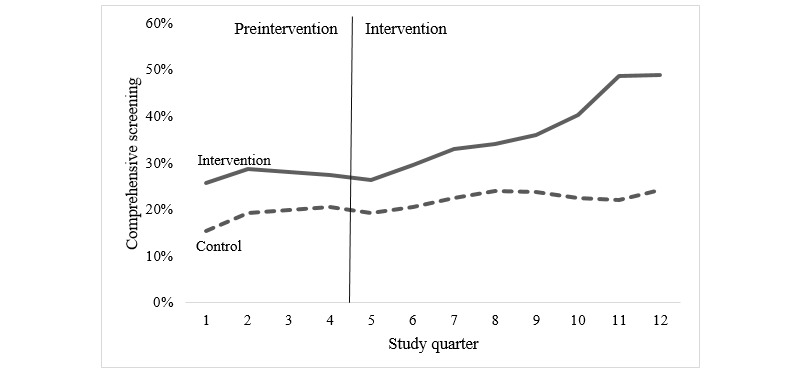
Comprehensive sexual health testing uptake among gay and bisexual men attending intervention and comparison sites.

In the 4 comparison sites, comprehensive testing uptake was lower overall than in the intervention sites but increased over time (Figure 3). During the 2-year period that the intervention was active in other sites, comprehensive testing for HIV and STIs increased from 19.25% (319/1657) to 24.26% (464/1913) among gay and bisexual men (*P*<.001). Compared with the prior 12 months, the SRR for these sites was 1.18 (95% CI 1.11-1.26, *P*<.001). Overall, although comprehensive testing for HIV and STIs increased across both intervention and comparison sites, the increase and difference between periods was greater for sites that received the intervention than for those that did not (SRR 1.12, 95% CI 1.10-1.14, *P*<.001).

#### Detection of HIV and Other Sexually Transmissible Infections

Finally, we explored changes in the detection of HIV and STIs between study and comparison sites. While there was a 46% increase in the detection of rectal chlamydia during the intervention, from a mean of 40.0 infections per quarter in the preperiod to 58.5 per quarter of the intervention (SRR 1.28, 95% CI 1.07-1.53, *P*=.007), there was a nonsignificant increase of 27% in comparison sites (SRR 1.17, 95% CI 0.99-1.37, *P*=.06). Similarly, while detection of urogenital chlamydia in clinics with the intervention was 44% higher during the intervention period than before (26.5 to 28.0 average per quarter, SRR 1.26, 95% CI 1.01-1.57, *P*=.04), there was no similar increase among comparison clinics (43.5 to 40.3 average per quarter, SRR 0.85, 95% CI 0.71-1.02, *P*=.09).

For gonorrhea, detection of rectal infections increased 45% from a mean of 25.4 to 35.0 per quarter during the intervention (SRR 1.27, 95% CI 1.01-1.61, *P*=.04) but was stable among comparison sites (SRR 1.14, 95% CI 0.97-1.34, *P*=.1). Pharyngeal diagnoses of gonorrhea were the one infection to increase between both study and comparison sites, rising from 23.8 to 46.0 per quarter in intervention sites (SRR 1.69, 95% CI 1.35-2.11, *P*<.001) and from 9.0 to 20.0 in comparison sites (SRR 2.04, 95% CI 1.42-2.94, *P*<.001). In the intervention sites there were no differences in diagnoses of infectious syphilis during the intervention (42.8 to 51.0, SRR 1.05, 95% CI 0.88-1.26, *P*=.6) or in HIV (13.5 to 13.0, SRR 0.75, 95% CI 0.52-1.05, *P*=.09), which was the same for comparison sites.

Of note, in both intervention and comparison sites, only 0.29% of syphilis tests (170/57,993) did not have sufficient information for classification as a new or previously treated infection.

## Discussion

### Principal Findings

The findings of this study suggest that integrated decision support software in general practice can improve sexual health care for gay and bisexual men. The implementation of software that facilitated routine feedback and prompted general practitioners to collect details on patient sexuality and offer comprehensive sexual health testing was associated with increased recording of sexual orientation among male patients and, among gay and bisexual men, increases in comprehensive sexual health testing in line with clinical guidelines. The observed increases in testing led to increased detection of infections with chlamydia and gonorrhea.

It is worth noting that testing for STIs increased across general practice settings that did and did not receive the intervention. Australia’s epidemiology of these infections has documented rising rates for years [2] and the governments of New South Wales and Victoria—the states in which this study took place—have both implemented numerous strategies aimed at increasing sexual health testing [29]. Nevertheless, the intervention appears to have contributed to higher rates of testing and diagnoses than would have otherwise taken place, suggesting a cumulative effective with other initiatives. Although promising, the observed increases were moderate, with the intervention demanding consistent energy to produce data reports and ensure that the software remained operational. Future analyses of integrated decision support software and quality improvement reports should consider the balance between costs and gains.

In spite of the increases in sexual orientation recording among male patients, at the end of the intervention period this variable remained unrecorded for the majority of men. Given the value of knowing sexual orientation for providing care beyond just sexual health [30], additional effort may be required to encourage the collection of this variable among clinicians in general practice. It is possible, however, that for some patients these details were recorded somewhere other than the “official” location in their file, which would not have been captured by our analysis.

The intervention appears to have encouraged greater completeness of testing among those engaged in sexual health care. The prompts, however, did not impact the overall uptake among gay and bisexual men, demonstrated by the stable proportion of men who received any form of sexual health testing. While testing may not have been required for some of the men who received no sexual health testing (ie, sexually inactive men, men who received sexual health care elsewhere), it would seem that the software was useful for capitalizing on existing testing opportunities but not necessarily for creating new ones. Different strategies to improve the overall offer of testing may be warranted, particularly among those not already engaged in sexual health care at a clinic.

While the intervention increased comprehensive testing, this was largely due to more samples being collected for chlamydia and gonorrhea; it had a lesser impact on testing for syphilis and no impact on HIV testing. This finding echoes earlier work assessing the effects of health promotion, which was associated with increased testing for chlamydia and gonorrhea but not syphilis or HIV [29]. It may be that some doctors are unaware of all the different samples required to effectively test for chlamydia and gonorrhea, with our findings echoing earlier work that found anal and throat swabs are among the most commonly missed [10]. It is also possible that patients are more likely to request a test for HIV than other STIs. Thus, the intervention’s impact in this domain highlights its usefulness for capitalizing on clinical encounters.

The observed increases in sexual orientation recording and comprehensive testing were gradual, due likely to the time it took for clinicians to become familiar with the software. Further, the time required to properly calibrate the software to each clinic’s technical infrastructure may also have hampered its usefulness in the intervention’s earlier days. These factors underscore the need for careful attention and routine follow-up to ensure that newly designed systems are functioning as expected.

### Limitations

It is possible that the changes in sexual orientation recording and comprehensive testing were due to some factor unrelated to this study’s intervention. This influence of external forces, however, was likely limited by the staggered intervention introduction, the fact that no significant trends were identified before the intervention, and the use of comparison sites. Further, we are unaware of any new clinical activities that occurred before or during the intervention period. It is also worth noting that study recruitment specifically targeted clinics providing care to gay and bisexual men based in major urban centers. As such, it is not possible to generalize these findings to other clinic or patient types, including those in rural or regional areas. Additional research is required to evaluate if this style of decision support software could similarly influence the delivery of sexual health care among other groups of patients.

A limitation of our analysis was the reliance on rectal swabs to identify gay and bisexual men in comparison sites, which while necessary may have actually diluted the intervention’s impact. This study was ecological in nature and, as such, required a body of analyses with nonoverlapping limitations. No one analysis proves the intervention’s impact but taken together they paint a complementary picture. Finally, noting the careful management required to ensure the intervention’s uptake in its early days, more research is required to assess the cost effectiveness of this kind of software and its sustainability over time.

### Conclusions

This study provides evidence that computerized clinical decision support systems can be effectively used in general practice to moderately improve sexual health clinical practice among gay and bisexual men. Further, as detecting these infections reduces the likelihood of onward transmission to sexual partners, these systems may have a part to play in reducing community prevalence of HIV and STIs.

## Appendix

Multimedia Appendix 1 Video guide of sexual health prompts and risk assessment dialogue introduced in general practice clinics via the PrimaryCare Sidebar.

Multimedia Appendix 2 Average annual trends and summary rate ratios in the quarterly proportions of male patients who had sexual orientation recorded, any HIV or other sexually transmissible infection tests, and comprehensive sexual health testing in the pre- and intervention periods.

## Abbreviations

ACCESS: Australian Collaboration for Coordinated Enhanced Sentinel Surveillance
STI: sexually transmissible infections
SRR: summary rate ratio
UNSW: University of New South Wales
