# Supplementary material for: Assessing the Impacts of Integrated Decision Support Software on Sexual Orientation Recording, Comprehensive Sexual Health Testing, and Detection of Infections Among Gay and Bisexual Men Attending General Practice: Observational Study
Source: JMIR Med Inform. 2018 Nov 6;6(4):e10808. doi: 10.2196/10808 (PMC6246964; doi:10.2196/10808)
Supplement: Multimedia Appendix 2 [file medinform_v6i4e10808_app2.pdf]

Multimedia Appendix 2. Average annual trends and summary rate ratios in the quarterly proportions of male patients who had: (i) sexual orientation recorded, (ii) any HIV or other STI test, and (iii) comprehensive sexual health testing* in the pre- and intervention periods

	Before period			Intervention period			Before vs intervention period		
	**Average annual trend**	**95% CI**	**p-trend**	**Average annual trend**	**95% CI**	**p-trend**	**Summary rate ratio**	**95% CI**	**p-trend**
**Sexual orientation**									
Overall (intervention)	1.00	0.99-1.03	0.3	1.02	1.01-1.03	<0.001	1.10	1.04-1.11	<0.001
Age (intervention)									
<30 years	1.03	0.97-1.10	0.314	1.08	1.06-1.10	<0.001	1.62	1.50-1.76	<0.001
30-49 ears	1.02	0.99-1.04	0.050	1.02	1.01-1.03	<0.001	1.10	1.04-1.14	<0.001
>50 years	1.00	0.98-1.04	0.396	1.02	1.01-1.03	0.002	1.07	1.02-1.13	0.008
HIV status (intervention)									
HIV positive	1.00	0.98-1.04	0.541	1.02	1.01-1.03	<0.001	1.11	1.07-1.15	<0.001
HIV negative	1.01	0.99-1.03	0.285	1.02	1.01-1.03	<0.001	1.27	1.24-1.30	<0.001
**Any HIV or other STI test**									
Overall (intervention)	1.00	0.97-1.02	0.834	1.00	0.99-1.00	0.983	0.97	0.94-1.00	0.156
Overall (comparison)	1.03	0.00-0.05	0.019	1.01	1.00-1.01	0.043	1.00	0.97-1.02	0.964
**Comprehensive testing**									
Overall (intervention)	1.01	0.96-1.07	0.5	1.09	1.08-1.11	<0.001	1.38	1.28-1.46	<0.001
Overall (comparison)	1.09	1.03-1.14	0.001	1.02	1.01-1.04	0.006	1.18	1.11-1.26	<0.001
Age (intervention)									
<30 years	1.09	0.90-1.31	0.4	1.11	1.07-1.16	<0.001	1.37	1.09-1.71	0.006
30-49 ears	1.02	0.96-1.09	0.6	1.09	1.07-1.11	<0.001	1.33	1.22-1.44	<0.001
>50 years	0.98	0.89-1.08	0.7	1.09	1.06-1.12	<0.001	1.41	1.24-1.60	<0.001
HIV status (intervention)									
HIV positive	1.06	0.97-1.14	0.2	1.11	1.08-1.14	<0.001	1.42	1.27-1.58	<0.001
HIV negative	0.99	0.93-1.05	0.7	1.08	1.06-1.10	<0.001	1.28	1.18-1.39	<0.001

*Tests for chlamydia (rectal, urogenital), gonorrhoea (rectal, pharyngeal), syphilis and, among men not know to be infected, HIV
